# Senolysis of gemcitabine‐induced senescent human pancreatic cancer cells

**DOI:** 10.1002/cnr2.2075

**Published:** 2024-04-25

**Authors:** Mohammad Mahbubul Hoque, Yuichi Iida, Hitoshi Kotani, Mamoru Harada

**Affiliations:** ^1^ Department of Immunology Shimane University Faculty of Medicine Izumo Shimane Japan

**Keywords:** Bcl‐xL, gemcitabine, pancreatic cancer, senescence, senolysis

## Abstract

**Introduction:**

Gemcitabine (GEM) is often used to treat pancreatic cancer. Many anti‐cancer drugs induce cancer cell death, but some cells survive after cell cycle arrest. Such a response to DNA damage is termed cellular senescence. Certain drugs, including the Bcl‐2‐family inhibitor ABT‐263, kill senescent cells; this is termed senolysis. In this study, we examined the therapeutic benefits of ABT‐263 in GEM‐induced senescence of human pancreatic cancer cells.

**Methods and Results:**

Of four pancreatic cancer cell lines (PANC‐1, AsPC‐1, CFPAC‐1, and PANC10.05), GEM induced senescent features in PANC‐1 and AsPC‐1 cells, including increases in the cell sizes and expression levels of mRNAs encoding interleukin (IL)‐6/IL‐8 and induction of β‐galactosidase. Successive treatment with GEM and ABT‐263 triggered apoptosis in PANC‐1 and AsPC‐1 cells and suppressed colony formation significantly. Senolysis of GEM‐induced senescent pancreatic cancer cells by ABT‐263 was triggered by a Bcl‐xL inhibitor, but not by a Bcl‐2 inhibitor, suggesting a central role for Bcl‐xL in senolysis. In a xenograft mouse model, combined treatment with GEM and ABT‐737 (an ABT‐263 analog exhibiting the same specificity) suppressed in vivo growth of AsPC‐1 significantly.

**Conclusion:**

Together, our results indicate that sequential treatment with GEM and senolytic drugs effectively kill human pancreatic cancer cells.

## 1 INTRODUCTION

Pancreatic cancer has a poor prognosis, and its incidence has increased continuously over the past several decades.^1^ Pancreatic cancer is the most therapy‐resistant cancer type. Existing treatment options include chemotherapy and radiation therapy; however, the results are unsatisfactory.^2^, ^3^, ^4^ Alternatively, gemcitabine (GEM), as a chemotherapeutic drug, is a useful drug for patients with pancreatic cancer. GEM is an analog of cytosine arabinoside that inhibits DNA replication, is often used to treat pancreatic cancer, either alone or in combination with other chemotherapeutics.^5^, ^6^, ^7^ A combination of 5‐fluorouracil, leucovorin, irinotecan, and oxaliplatin (FOLFIRINOX), increases the survival of patients with high‐grade pancreatic cancer to a greater extent than GEM, although its toxicity is problematic.^8^, ^9^ GEM is the main chemotherapeutic drug for patients with pancreatic cancer; that said, GEM in combination with other drugs might be more effective in treating pancreatic cancer.

Recently, senescence has received much attention in anti‐aging and age‐related disease studies.^10^, ^11^ Senescence is a cellular stress response triggered by aging, aberrant oncogene activation, and DNA damage.^12^, ^13^ Although anti‐cancer drugs are generally used to kill cancer cells, some cells survive via DNA damage responses and acquire features of senescence: growth arrest, size enlargement, expression of β‐galactosidase, and the production of proinflammatory cytokines and other factors.^12^, ^13^, ^14^, ^15^ The production of proinflammatory cytokines and other factors is termed the senescence‐associated (SA) secretory phenotype (SASP)^16^, ^17^, ^18^, ^19^, ^20^; recent studies suggest that senescent cancer cells showing SASP trigger cancer recurrence and metastasis.^21^ Thus, targeting therapy‐induced senescent cancer cells may prevent recurrence and metastasis after anti‐cancer chemotherapy.^22^

Recently, several drugs have been revealed to target such senescent cells; the selective elimination of senescent cells is termed senolysis.^23^, ^24^, ^25^ Several drugs have potential senolytic effects.^26^ ABT‐263 (navitoclax), an oral inhibitor of Bcl‐2, Bcl‐xL, and Bcl‐w, is a representative drug that can lyse senescent cells.^27^, ^28^ In this regard, we previously reported that ABT‐263 and its analog ABT‐737 had senolytic activity against drug‐induced senescent human breast and lung cancer cells both in vitro and in vivo.^29^, ^30^ We also found that GEM could induce senescence in some human pancreatic cancer cell lines. These findings led us to test whether senolytic drugs could effectively induce cell death in GEM‐treated senescent human pancreatic cancer cells.

## 2 MATERIALS AND METHODS

### 2.1 Cell lines and reagents

Three human pancreatic cancer cell lines (AsPC‐1, CFPAC‐1, and PANC10.05) were purchased from the ATCC. PANC‐1 cells were provided by Dr. K. Takenaga (Shimane University). All cell lines carried mutated KRAS. In addition, the PANC‐1 and PANC10.05 cell lines carry mutated p53, whereas AsPC‐1 and CFPAC‐1 carry wild‐type p53. All cell lines were maintained in DMEM (Sigma‐Aldrich) supplemented with 10% fetal calf serum (Biosera) and 20 μg/mL gentamicin (Sigma‐Aldrich). GEM was purchased from ChemScience. Both ABT‐263 and ABT‐737 were obtained from Active Biochemicals Co. Ltd. ABT‐199 was purchased from ChemieTek. A‐1331852 was obtained from Selleck. All three drugs were diluted in dimethylsulfoxide (DMSO).

### 2.2 Cell viability assay

Cell viability was evaluated using the Cell Counting Kit‐8 (CCK‐8; Nacalai Tesque). Cancer cells (2 × 10^4^ cells/well) were seeded in volumes of 100 μL into 96‐well plates. After adding the indicated doses of reagents, cells were cultured for 2 or 4 days. Then, 10 μL of WST‐8 solution was added and the cells were incubated for an additional 3 h. The absorbances at 450 nm were measured using a microplate reader (Beckman Coulter). The relative cell viabilities (%) compered untreated control are shown.

### 2.3 Microscopic analysis

Cancer cells were seeded into six‐well plates and cultured for 2 and 4 days with the indicated doses of GEM. Photographs were taken using cameras mounted on Nikon (ECLIPSE Ts2).

### 2.4 Immunoblotting

Harvested cells were lysed with radio‐immunoprecipitation assay buffer (Fujifilm Wako Pure Chemical) containing cocktails inhibiting protease and phosphatase (Nacalai Tesque). Proteins were electrophoresed on sodium dodecyl sulfate pol yac rylamide gel electrophoresis gels and transferred to polyvinylidene fluoride membranes. After blocking, the membranes were incubated with the following antibodies: anti‐Bcl‐2 (clone 100, #658701; BioLegend); anti‐Bcl‐xL (clone 54H6, #2764; Cell Signaling Technology); anti‐Mcl‐1 (#54535; Cell Signaling Technology); anti‐p21^Cip1/Waf1^ (p21) (#2947; Cell Signaling Technology); anti‐p16^Ink4a^ (p16) (SPC‐1280; StressMarq Biosciences); and anti‐β‐actin (#622102; BioLegend). Thereafter, the membranes were incubated at room temperature for 60 min with goat anti‐rabbit or horse anti‐mouse horseradish peroxidase‐conjugated secondary antibody (#7074 and #7076; Cell Signaling Technology). Bands were visualized by an Amersham ImageQuant 800 Biomolecular Imager (Global Life Sciences Technologies Japan).

### 2.5 Quantitative real‐time polymerase chain reaction (qPCR)

Total RNAs were extracted from cells using TRIzol (Invitrogen) and complementary DNA synthesized employing the ReverTra Ace qPCR RT Master Mix with gNDA Remover (Toyobo). We performed qPCR using the Thunderbird qPCR Mix (Toyobo). The expression levels of target genes were normalized to that of β‐actin by the comparative 2^−ΔΔCT^ method. The primer sets were:

IL‐6 F: 5′‐aaagaggcactggcagaaaa‐3′, IL‐6 R: 5′‐tttcaccaggcaagtctcct‐3′,

IL‐8 F: 5′‐tctggcaaccctagtctgct‐3′, IL‐8 R: 5′‐gcttccacatgtcctcacaa‐3′,

β‐actin F: 5′‐ttgccgacaggatgcagaa‐3′, and β‐actin R: 5′‐gccgatccacacggagtact‐3′.

### 2.6 Evaluation of cell size and β‐galactosidase expression

The expression of SA β‐Gal was evulated by the Cellular Senescence Detection Kit (DOJINDO). In brief, cancer cells (2 × 10^5^ cells/well) were seeded into 12‐well plates in 2 mL volumes with the indicated doses of GEM. After 3 days, the plates were washed twice with Hanks' balanced salt solution (Sigma‐Aldrich) and incubated with bafilomycin A1 for 1 h, subsequently with SPiDER‐β‐Gal for 30 min at 37°C. After harvesting, cell size and SA β‐galactosidase expression were assayed by CytoFLEX flow cytometry (Beckman Coulter) and analyzed using CytExpert software (Beckman Coulter).

### 2.7 Apoptosis

Cancer cells (2 × 10^4^/well) were seeded (2 mL) into six‐well plates and GEM and/or ABT‐263 was added. ABT‐263 was added 2 days later than GEM. Four days after GEM/ABT‐263 addition, the cells were harvested and washed with phosphate‐buffered saline (PBS). Thereafter, the harvested cells were stained with FITC‐conjugated annexin V and propidium iodide (PI) (BioVision), and subjected to CytoFLEX flow cytometry and analyzed by CytExpert software.

### 2.8 Colony formation assay

Cells were seeded into six‐well plates and cultured with various levels of GEM and/or ABT‐263. GEM was added at culture commencement, and ABT‐263 was added 2 days later. On day 4, the medium was changed, and culture proceeded without drugs. On day 14, the cells were fixed by methanol and stained with 0.05% crystal violet to visualize colonies. After taking photograph, the colony numbers were counted.

### 2.9 In vivo xenograft model

Female 6‐week‐old nude BALB mice (CLEA) were injected subcutaneously in the right flank with 2 × 10^6^ AsPC‐1 cells and Matrigel (BD Biosciences) at a 1:1 ratio and a volume of 100 μL. Seven days later, the mice were randomly divided into four groups and intraperitoneally injected with GEM (50 mg/kg) on days 7, 11, and 15 and/or ABT‐737 (35 mg/kg) on days 8, 9, 12, 13, 16, and 17. As controls for GEM and ABT‐737, the same volumes of PBS and DMSO were injected intraperitoneally, respectively. The tumor volumes and body weights were measured twice weekly. The tumor volume was calculated as follows: Volume (mm^3^) = (length × width^2^) ÷ 2. On day 21, all mice were alive, and were euthanized.

### 2.10 Statistical analysis

Group means were compared using analysis of variance, followed by the Tukey–Kramer test (more than two groups). Significance was determined at *p* < 0.05.

## 3 RESULTS

### 3.1 Effects of GEM on four human pancreatic cancer cell lines

**FIGURE 1:**
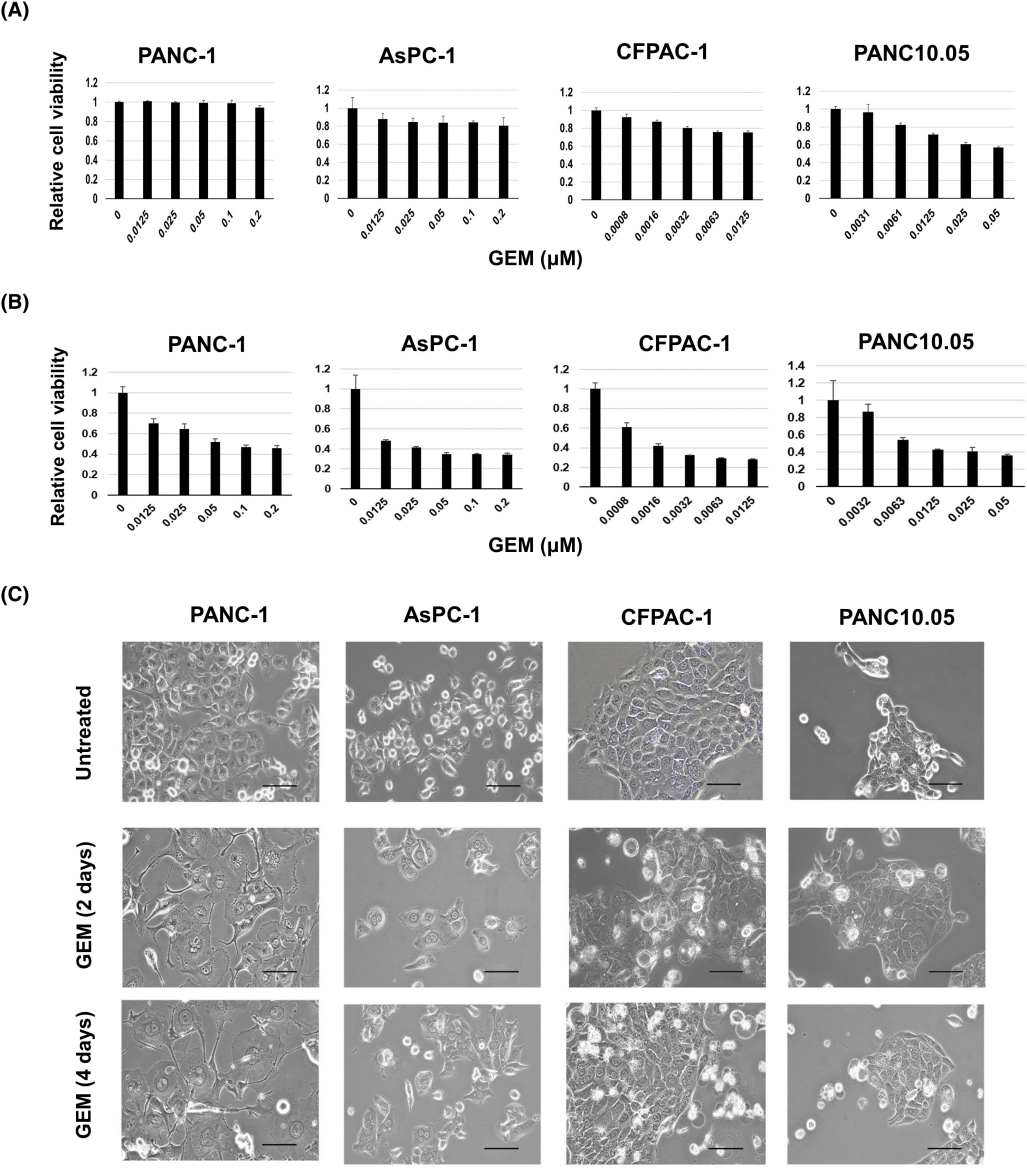
Effects of gemcitabine (GEM) on four human pancreatic cancer cell lines. Four pancreatic cancer lines were cultured with the indicated doses of GEM for 2 days (A) and 4 days (B), and cell viability was determined using a Cell Counting Kit‐8 (CCK) assay. Data are means ± standard deviation (SD). (C) Four cancer cell lines were cultured in the presence of GEM (0.0125 μM) for 2 and 4 days and photographed. Magnification ×20; Scale bar 100 μm.

We examined the effects of GEM on the viability of four pancreatic cancer cell lines cultured for 2 or 4 days. By day 2, GEM decreased the viability of PANC10.05 cells, and those of AsPC‐1 and CFPAC‐1 cells to lesser extents (Figure 1A). GEM did not appear to affect PANC‐1 cells. However, by day 4, all four cell lines decreased their viability in dose‐dependent manners (Figure 1B). The effects of GEM varied; CAPAC‐1 cells were most sensitive to GEM, followed by PANC10.05, PANC‐1, and AsPC‐1 cells. Notably, when the four cell lines were treated with 0.0125 μM GEM for 2 and 4 days, the size of the PANC‐1 cells increased remarkably without cell death (Figure 1C). Although the size of the AsPC‐1 cells also increased to a degree, no such change was observed in CFPAC‐1 and PANC10.05 cells.

### 3.2 GEM induces senescence of PANC‐1 and AsPC‐1 cells

**FIGURE 2:**
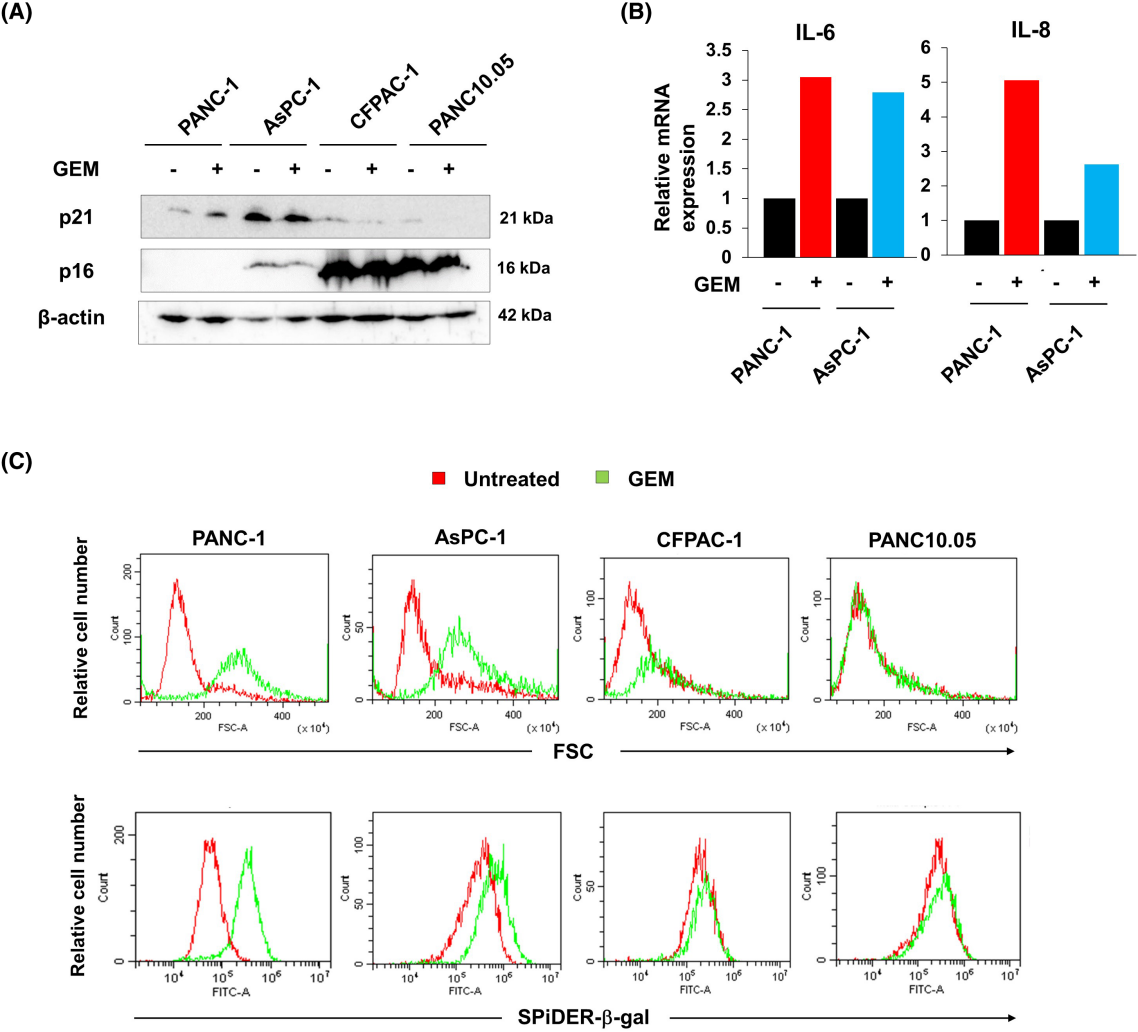
Gemcitabine (GEM) induces senescent features in PANC‐1 and AsPC‐1 cells. (A) Four pancreatic cancer cell lines were cultured with GEM (0.0125 μM) for 48 h. The levels of the p21 and p16 proteins were determined via immunoblotting. β‐Actin was used as the loading control. (B) PANC‐1 and AsPC‐1 cells were cultured with GEM (0.0125 μM). Total RNAs were extracted and real‐time polymerase chain reaction (qPCR) was performed. (C) Four cell lines were cultured in 12‐well plates with GEM (PANC‐1 and AsPC‐1, 0.0125 μM; CFPAC‐1, 0.0008 μM; PANC10.05, 0.0032 μM) for 3 days. Using the Cellular Senescence Detection Kit, SA‐β‐gal was stained. Forward side scattering (FSC) and the SA SPiDER‐β‐gal levels were measured by flow cytometry. The red and green lines are the data for the untreated and GEM‐treated groups, respectively.

Increased cell size is a feature of senescence.^14^, ^15^ Therefore, we tested whether GEM induced senescence. Generally, cell growth arrest is induced by overexpression of p21 and p16.^31^ GEM increased the expression of p21 in PANC‐1 cells, but AsPC‐1 cells constitutively expressed p21 in a manner not influenced by GEM (Figure 2A). The other two cell lines (CFPAC‐1 and PANC10.05) expressed very little p21. PANC‐1 cells were negative for p16, and the other three cell lines exhibited no change in p16 expression after GEM treatment. CFPAC‐1 and PANC10.05 cells constitutively expressed p16 at high levels. Senescent cells produce several inflammatory cytokines and other factors.^16^, ^17^, ^18^, ^19^, ^20^ Real‐time PCR showed that the levels of mRNAs encoding IL‐6 and IL‐8 in PANC‐1 and AsPC‐1 cells were increased by GEM treatment (Figure 2B). Next, we examined the effects of GEM on cell size by evaluating the forward side scattering (FSC). The FSC of PANC‐1 and AsPC‐1 cells shifted to the right after GEM treatment for 3 days, indicating that their cell sizes had increased (Figure 2C). Such GEM‐induced changes were also slightly observed in CFPAC‐1cells, but not in the PANC10.05 cell line. We also examined SA β‐galactosidase expression, which is also increased in senescent cells^14^, ^15^; GEM treatment for 3 days apparently increased the SA β‐galactosidase expression in PANC‐1 and AsPC‐1 cells. No such change was observed in CFPAC‐1 and PANC10.05 cells. Considering the GEM‐induced changes in cell appearance, size, IL‐6/IL‐8 mRNA expression, and SA β‐galactosidase expression, these results indicate that GEM induced senescence in PANC‐1 and AsPC‐1 cells.

### 3.3 Combined effects of GEM and ABT‐263 on the viability of four pancreatic cell lines

**FIGURE 3:**
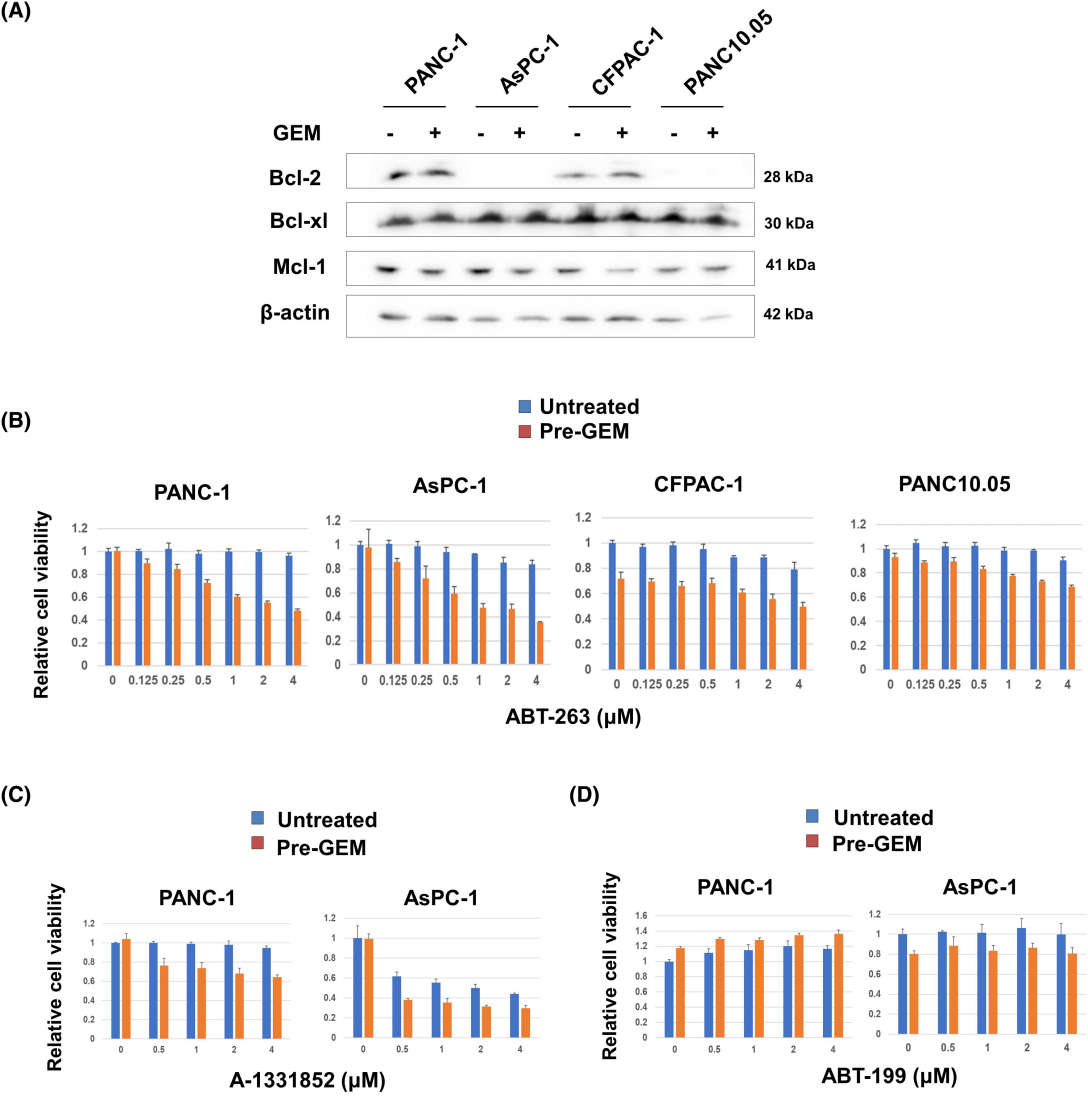
Combined effects of gemcitabine (GEM) and Bcl‐2 family inhibitors on cancer cells. (A) Four pancreatic cancer cell lines were cultured with GEM (0.0125 μM) for 2 days and the levels of the Bcl‐2, Bcl‐xL, and Mcl‐1 proteins were determined; β‐Actin was used as the loading control. (B) The four lines were cultured with GEM (PANC‐1 and AsPC‐1, 0.0125 μM; CFPAC‐1, 0.0008 μM; PANC10.05, 0.0032 μM) for 2 days and, after harvesting, cultured with the indicated doses of ABT‐263. After 2 days, cell viability was determined using a CCK‐8 assay. PANC‐1 and AsPC‐1 cells were cultured with GEM (0.0125 μM) for 2 days. After harvesting, cells were cultured with (C) A‐1331852 or (D) ABT‐199. After 2 days, cell viability was determined using a CCK‐8 assay. Data are means ± SD. All experiments were performed in triplicate and repeated three times.

Recently, ABT‐263 was shown to be a senolytic drug, that is, lethal to senescent cells.^23^, ^24^, ^25^ We previously found that senescent breast and lung cancer cells were lysed by ABT‐263.^29^, ^30^ Therefore, we explored whether GEM‐treated pancreatic cancer cells could be lysed by ABT‐263. First, we checked the expression levels of anti‐apoptotic Bcl‐2 family proteins. Both AsPC‐1 and PANC10.05 were negative for Bcl‐2, and GEM did not affect Bcl‐2 the expression of Bcl‐2 in either cell line (Figure 3A). GEM tended to decrease the Mcl‐1 expression of CFPAC‐1 cells. Overall, GEM exerted no clear effect on the levels of anti‐apoptotic Bcl‐2 family proteins.

Next, we examined the effects of ABT‐263 on untreated and GEM‐pretreated pancreatic cancer cells. ABT‐263 diminished the viability of GEM‐pretreated PANC‐1 and AsPC‐1 cells in dose‐dependent manners (Figure 3B), but no such effect was apparent for GEM‐pretreated CFPAC‐1 and PANC10.05 cells. A‐1331852, a Bcl‐xL inhibitor, decreased the viability of GEM‐pretreated PANC‐1 and AsPC‐1 cells (Figure 3C), whereas ABT‐199, a Bcl‐2 inhibitor, did not (Figure 3D).

### 3.4 Combined effects of GEM and ABT‐263 on colony formation

**FIGURE 4:**
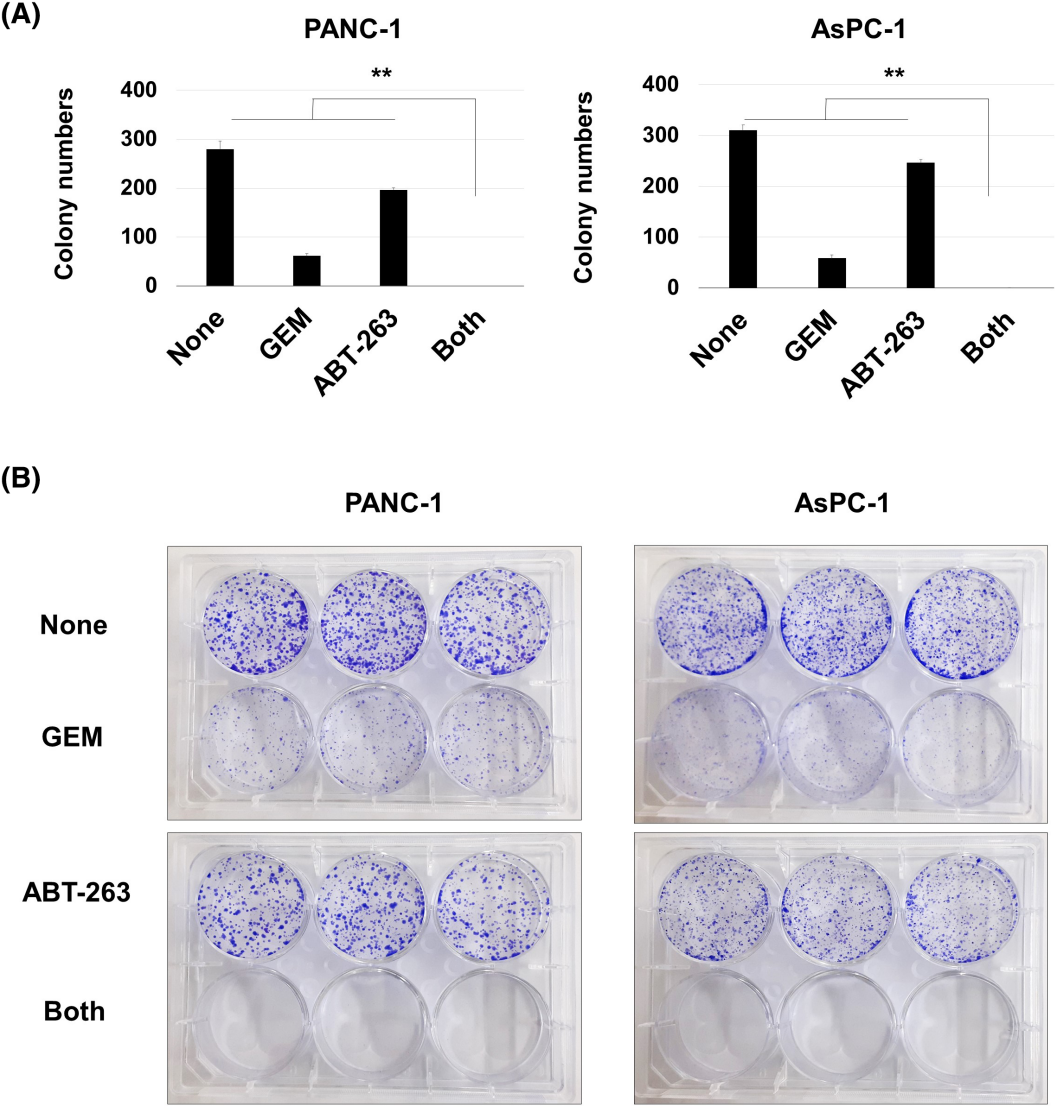
Combined effects of gemcitabine (GEM) and ABT‐263 on colony formation in pancreatic cancer cells. (A) PANC‐1 and AsPC‐1 cells were cultured with GEM (0.0125 μM) and/or ABT‐263 (2 μM) for 3 days. The medium was changed and cells were cultured in the absence of any drug for a further 2 weeks; colonies were visualized using crystal violet staining. Data are means ± SD. The experiments were performed in triplicate and repeated three times. Statistical significance was evaluated using the Tukey–Kramer test (**p < 0.01). (B) Representative results are shown.

Next, we tested the combined effects of GEM and ABT‐263 on the abilities of forming colonies using PANC‐1 and AsPC‐1 cells. They were cultured with either or both GEM and ABT‐263 for 72 h. After the removal of the drugs, culture continued for a further 2 weeks. GEM significantly suppressed colony numbers, whereas ABT‐263 was less effective (Figure 4A). When the drugs were combined, no colonies formed (**p < 0.01; the both group vs. the other groups). Representative results are shown in Figure 4B.

### 3.5 Senolysis of GEM‐induced senescent PANC‐1 and AsPC‐1 cells by ABT‐263

**FIGURE 5:**
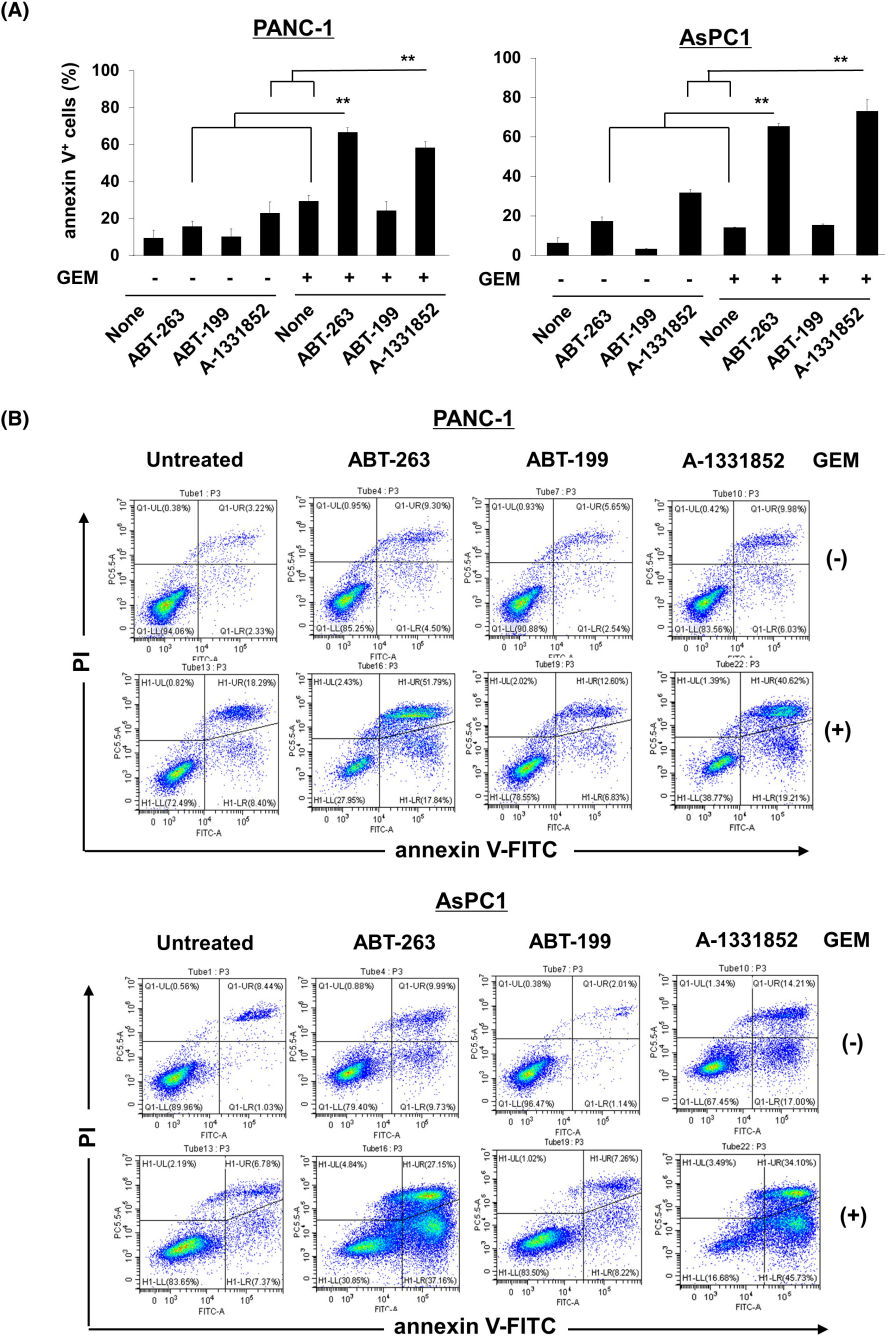
ABT‐263‐induced senolysis in cancer cells that entered senescence after gemcitabine (GEM) treatment. (A) PANC‐1 and AsPC‐1 cells were cultured with GEM (0.0125 μM) for 2 days and ABT‐263, ABT‐199, or A1331852 (2 μM) was then added, followed by culture for a further 2 days. After staining with annexin V‐FITC and propidium iodide (PI), cells were subjected to flow cytometry. Data are means ± SD. All experiments were performed in triplicate and repeated twice. Statistical significance was evaluated using the Tukey–Kramer test (**p < 0.01). (B) Representative results are shown.

Next, we used flow cytometry to determine whether ABT‐263 induced senolysis in senescent PANC‐1 and AsPC‐1 cells. These cells were cultured at the presence of GEM for 48 h, and ABT‐263, ABT‐199, or A‐1331852 was added, followed by culture for a further 48 h. Compared to treatment with either GEM or ABT‐263, combined treatment significantly increased the proportions of apoptotic (annexin V^+^) PANC‐1 and AsPC‐1 cells (Figure 5A) (**p < 0.01; combination group vs. monotherapy groups). Apoptosis was enhanced similarly when GEM was combined with A‐1331852 (**p < 0.01; combination group vs. monotherapy groups), but not with ABT‐199. Representative data are shown in Figure 5B.

### 3.6 Combined effects of GEM and ABT‐737 on in vivo growth of AsPC‐1

**FIGURE 6:**
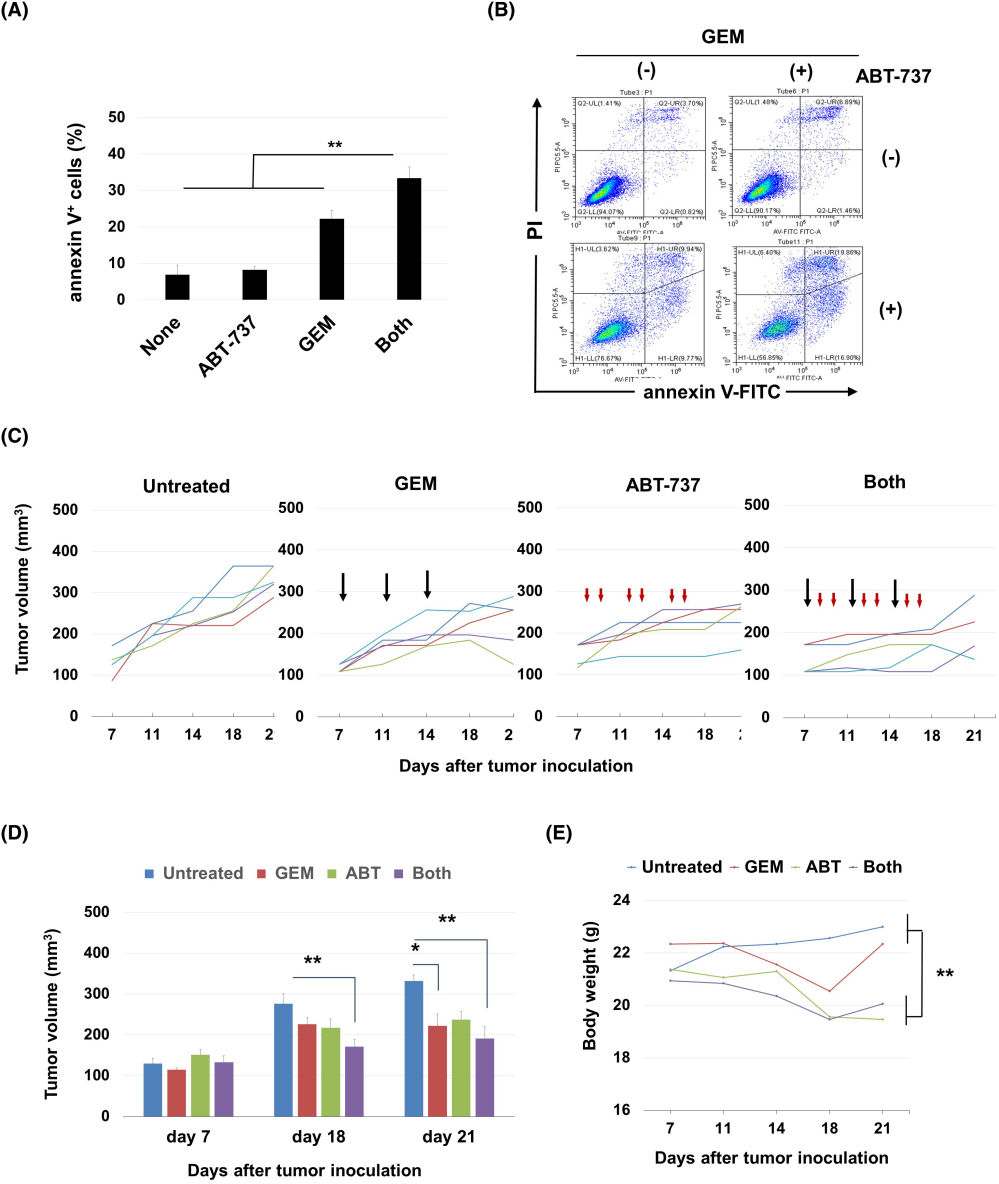
Effect of combined gemcitabine (GEM) and ABT‐737 on in vivo growth of AsPC‐1 cells. (A) AsPC‐1 cells were cultured with GEM (0.0125 μM) for 2 days and then ABT‐737 (2 μM) was added, followed by further culture for 2 days. Harvested cells were stained with annexin V‐FITC and propidium iodide (PI) and analyzed via flow cytometry. Data are means ± SD. Statistical significance was evaluated using the Tukey–Kramer test (**p < 0.01). The experiments were performed in twice. (B) Representative results are shown. (C) Female nude BALB mice were subcutaneously injected in the right flank with 2 × 10^6^ AsPC‐1 cells. On day 7, the mice were divided into four groups and intraperitoneally injected with GEM (50 mg/kg) (black arrows) on days 7, 11, and 15 and ABT‐737 (35 mg/kg) (red arrowheads) on days 8, 9, 12, 13, 16, and 17. Each line indicates the tumor volume of one mouse. (D) Histograms of tumor volumes. (E) Body weights on day 21. Statistical significance was evaluated using the Tukey–Kramer test (*p < 0.05; **p < 0.01).

Finally, we examined the effects of a senolytic drug on GEM‐induced senescent pancreatic cancer cells in vivo. As ABT‐263 is given orally,^27^, ^28^ we used its homolog ABT‐737, which has the same specificity but is systemically administered.^27^ In vitro, the combined treatment with GEM and ABT‐737 significantly increased the proportion of apoptotic AsPC‐1 cells (Figure 6A) (**p < 0.01; the both group vs. the other groups). A representative result is shown in Figure 6B. Next, we evaluated the combined effects of GEM and ABT‐737 on the growth of AsPC‐1 using an in vivo xenograft model (Figure 6C,D). The combined therapy with GEM and ABT‐737 significantly retarded the tumor growth on days 18 and 21 (**p < 0.01; the both group vs. the untreated group). The monotherapy with GEM significantly retarded the tumor growth on day 21 (*p < 0.05; the untreated group vs the GEM‐treated group). The photographs of tumors on day 21 are shown in Figure S1. ABT‐737 with or without GEM reduced body weight (Figure 6E) (**p < 0.01; the untreated or GEM‐treated groups vs. the ABT‐treated or the combination group). This effect on body weight loss was principally attributable to ABT‐737; however, all mice tolerated the combined therapy and survived.

## 4 DISCUSSION

Recently, senescence has received a lot of attention in anti‐aging and age‐related disease studies.^10^, ^11^ However, this study focused on drug‐induced senescent cancer cells. In this regard, we recently reported that the CDK4/6 inhibitor, abemaciclib, induces senescence in human breast cancer cells, which are effectively lysed by ABT‐263,^29^ and that the anti‐folate drug, pemetrexed, induces senescence in human lung cancer cells, which are effectively lysed by ABT‐737.^30^ Based on these results, we investigated GEM‐induced senescent pancreatic cancer cells in this study.

Pancreatic cancer is the most therapy‐resistant cancer type and has a poor prognosis; its incidence has increased continuously over the past several decades.^1^ Although the therapeutic efficacy of chemotherapy is unsatisfactory,^2^, ^3^, ^4^ GEM is the main treatment of pancreatic cancer. Therefore, we searched for new treatments targeting GEM‐treated pancreatic cancer cells. In this study, we revealed that, of four human pancreatic cancer cell lines, GEM induced senescent features in PANC‐1 and AsPC‐1, leading to growth arrest, increased cell size, enhanced the expression of mRNAs encoding IL‐6/IL‐8 mRNA, and induction of β‐galactosidase (Figure 2). Generally, SA growth arrest is induced by p21 and/or p16^31^; however, GEM increased the expression of p21 only in PANC‐1 cells. PANC‐1 cells were p16‐negative and GEM did not change the p16 expression level of AsPC‐1 cells. However, a low dose of GEM (0.0125 μM) decreased the cell numbers without death, indicating that GEM induced growth arrest in these cells.

Two studies reported that GEM induced senescence in human pancreatic cancer cells. Song et al.^32^ reported that GEM rendered GEM‐resistant Miapaca‐2 and PANC‐1 cell lines senescent, but not the GEM‐sensitive L3.6pl human pancreatic cell line. Jaber et al.^33^ found that, of 11 human pancreatic cancer cell lines, PANC‐1, and Capan2 cells developed features of senescence in response to GEM. We used four pancreatic cancer cell lines, of which three (AsPC‐1, CFPAC‐1, and PANC10.05) have not been examined previously. GEM induced senescence in PANC‐1 and AsPC‐1 cells. Given the previous data^32^, ^33^ and our present findings, it is apparent that GEM‐induced senescence is preferentially induced in pancreatic cancer cell lines, suggesting that cancer cells that undergo therapy‐induced senescence survive when exposed to anti‐cancer drugs.

We previously reported that Bcl‐xL is a key event in human pancreatic cancer cell apoptosis induced by the TNF‐related apoptosis‐inducing ligand.^34^ Although the cancer type and anti‐cancer drug differed, Saleh et al.^25^ reported that senescent human breast and lung cancer cells pretreated with etoposide or doxorubicin were lysed by ABT‐263, which interfered with the Bcl‐xl/Bax interaction. Thus, Bcl‐xL is a key molecule in terms of the apoptosis/senolysis of GEM‐induced, senescent human pancreatic cancer cells. On the other hand, A‐1331852 induced more apoptotic events than ABT‐263 (Figure 5). Although we have no clear explanation for this result, their different specificities could account for their specificity; A‐1331852 specifically inhibits Bcl‐xL, whereas ABT‐263 inhibits Bcl‐2/xL/w.

We first used cell viability assays to examine the effects of combined GEM and ABT‐263. However, cell viability is affected by both cell growth and death. An apoptosis assay better explores senolysis. Apoptosis increased in PANC‐1 and AsPC‐1 cell lines pretreated with GEM followed by ABT‐263 or A1331852 (Figure 5) or ABT‐737 (Figure 6A, B). We also examined the combined effects of GEM and ABT‐263 on colony formation (Figure 4). GEM alone significantly suppressed colony formation in both cell lines, whereas the combination eliminated colony formation completely. These results suggest the effectiveness of sequential therapy: first with GEM and subsequently with senolytic drugs.

In the xenograft model, we used ABT‐737 rather than ABT‐263, as they have identical specificity,^27^ whereas the former can be systemically administered. We previously reported that ABT‐737 exhibited antitumor effects against drug‐induced human breast and lung cancer cells both in vitro and in vivo when combined with a CDK4/6 inhibitor and pemetrexed, respectively.^29^, ^30^ In this study, combined treatment with GEM and ABT‐737 retarded the in vivo tumor growth of AsPC‐1 cells to a greater extent than either GEM or ABT‐737 monotherapy (Figure 6). We performed in vivo experiments to test the AsPC‐1 combinations because a prior study reported combined effects of GEM and ABT‐263 against PANC‐1 cells.^33^ We did not perform in vivo experiments using ABT‐263 because ABT‐263 is administered orally and our previous study showed that oral ABT‐263 was ineffective in nude mice that were inoculated with human prostate cancer cells subcutaneously.^35^ Therefore, we used ABT‐737 in in vivo experiments. Together, these results suggest that senolysis of GEM‐induced senescent pancreatic cancer cells could be achieved in vivo.

We demonstrated that PANC‐1 and AsPC‐1 pancreatic cancer cells exhibited senescent features when exposed to low doses of GEM. Importantly, these cells were effectively lysed by senolytic drugs. Although we planned to perform in vivo experiments using PANC‐1 cells, Jaber et al. reported in vivo senolysis of PANC‐1.^33^ Therefore, we focused on AsPC‐1 cells. Nevertheless, this is the first report of senolysis of AsPC‐1 cells both in vitro and in vivo. However, there are several limitations to our study. First, GEM administration might induce senescence in normal cells in vivo, and we did not examine normal tissues. Second, we have not examined the induction of senescence in human pancreatic cancer tissues of patients who received GEM treatment. Third, ABT‐263 was reported to induce thrombocytopenia.^36^ We simply evaluated systemic adverse events using body weight. In this regard, a recent report has shown that galacto‐conjugated ABT‐263 can increase senolytic specificity, while reducing platelet toxicity.^37^ Furthermore, studies are needed to ascertain the effectiveness of the senolytic strategy of GEM‐induced senescent pancreatic cancer cells.

## AUTHOR CONTRIBUTIONS

**Mohammad Mahbubul Hoque:** Conceptualization (lead); data curation (lead); funding acquisition (lead); investigation (lead); writing – original draft (lead). **Yuichi Iida:** Data curation (supporting); formal analysis (supporting); investigation (supporting); writing – review and editing (equal). **Hitoshi Kotani:** Data curation (supporting); formal analysis (supporting); methodology (supporting); writing – review and editing (equal). **Mamoru Harada:** Conceptualization (lead); data curation (supporting); formal analysis (lead); funding acquisition (lead); investigation (lead); methodology (supporting); resources (lead); supervision (lead); writing – review and editing (lead).

## FUNDING INFORMATION

JSPS KAKENHI (Grant Number 21K07177) to M.H. and JST SPRING (Grant Number JPMJSP2155) to M.M.H.

## CONFLICT OF INTEREST STATEMENT

The authors declare no conflicts of interest.

### ETHICS STATEMENT

Animal experiments were approved by the Animal Care and Use Committee of Shimane University (approval no. IZ4‐40).

## Supporting information


**Figure S1.** Photographs of tumors on day 21 after AsPC‐1 inoculation.

## Data Availability

Data sharing is not applicable to this article as no new data were created or analyzed in this study.
