# Supplementary figures and images for: Senolysis of gemcitabine‐induced senescent human pancreatic cancer cells
Source: Cancer Rep (Hoboken). 2024 Apr 25;7(4):e2075. doi: 10.1002/cnr2.2075 (PMC11044911; doi:10.1002/cnr2.2075)

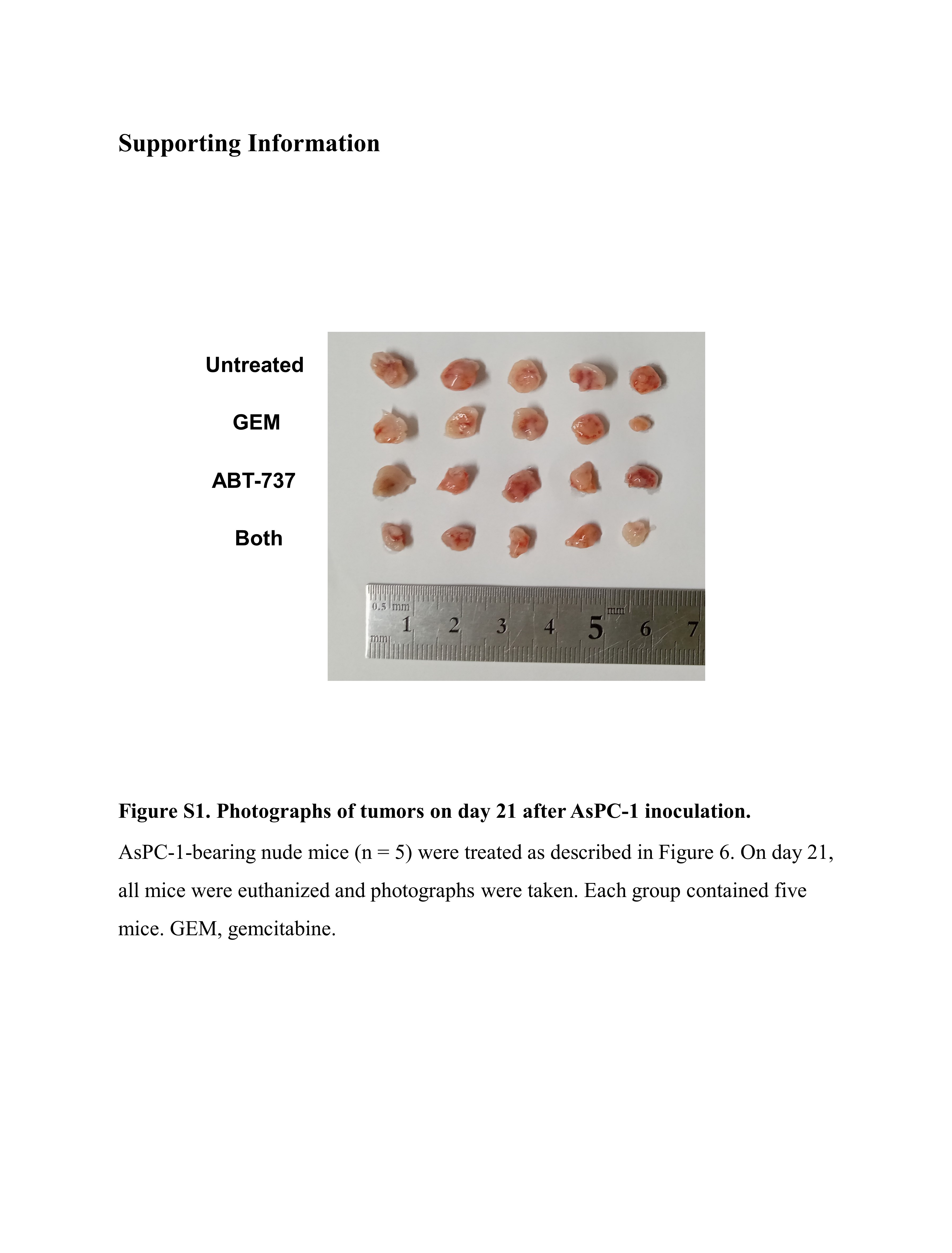

Supplement: Supplementary file 1 — Figure S1. Photographs of tumors on day 21 after AsPC‐1 inoculation. [file CNR2-7-e2075-s001.jpg]
